# Diagnosis and management of multiple sclerosis: MRI in clinical practice

**DOI:** 10.1007/s00415-020-09930-0

**Published:** 2020-05-29

**Authors:** Valentina Tomassini, Audrey Sinclair, Vijay Sawlani, James Overell, Owen R. Pearson, Julie Hall, Joe Guadagno

**Affiliations:** 1 Department of Neurosciences, Imaging and Clinical Sciences, Institute of Advanced Biomedical Technologies (ITAB), University of Chieti-Pescara “G. d’Annunzio”, Chieti, Italy; 2 Division of Psychological Medicine and Clinical Neurosciences, Cardiff University School of Medicine, Cardiff, UK; 3 Department of Neuroradiology, St Georges Hospital, London, UK; 4 Queen Elizabeth Hospital, Birmingham, UK; 5 University of Glasgow, Glasgow, UK; 6 Morriston Hospital, Swansea and Neath Port Talbot Hospitals, Port Talbot, UK; 7 Royal Victoria Infirmary, Newcastle, UK; 8 Newcastle Hospitals, Newcastle, UK

**Keywords:** Multiple sclerosis, MRI, Clinical practice, Diagnosis, Monitoring, Disease-modifying treatments

## Abstract

**Background:**

Recent changes in the understanding and management of multiple sclerosis (MS) have increased the role of MRI in supporting diagnosis and disease monitoring. However, published guidelines on the use of MRI in MS do not translate easily into different clinical settings and considerable variation in practice remains. Here, informed by published guidelines for the use of MRI in MS, we identified a clinically informative MRI protocol applicable in a variety of clinical settings, from district general hospitals to tertiary centres.

**Methods:**

MS specialists geographically representing the UK National Health Service and with expertise in MRI examined existing guidelines on the use of MRI in MS and identification of challenges in their applications in various clinical settings informed the formulation of a feasible MRI protocol.

**Results:**

We identified a minimum set of MRI information, based on clinical relevance, as well as on applicability to various clinical settings. This informed the selection of MRI acquisitions for scanning protocols, differentiated on the basis of their purpose and stage of the disease, and indication of timing for scans. Advice on standardisation of MRI requests and reporting, and proposed timing and frequency of MRI scans were generated.

**Conclusions:**

The proposed MRI protocol can adapt to a range of clinical settings, aiding the impetus towards standardisation of practice and offering an example of research-informed service improvement to support optimisation of resources. Other neurological conditions, where a gap still exists between published guidelines and their clinical implementation, may benefit from this same approach.

## Introduction

Magnetic resonance imaging (MRI) is routinely used in clinical practice to detect and monitor inflammatory lesions in patients with multiple sclerosis (MS) [1]. Diagnostic criteria, as well as disease-modifying treatments (DMTs), have more recently increased the demand for MRI scans to allow early diagnosis and monitoring of treatment safety and efficacy [2, 3]. This has increased the burden on equipment and healthcare professionals, requiring rationalisation of resources.

Published guidelines on the use of MRI in MS exist [4, 5]. While these guidelines provide comprehensive recommendations, they do not translate easily into different clinical settings and considerable variation in practice remains [6–8]. Indeed, wide variations exist in the MRI acquisition protocols and timing of scanning, as well as in the reporting of images [9]. This makes within-patient, as well as between-centre comparisons difficult, delaying diagnosis and affecting treatment decisions. This variability underlines the need for further direction on the implementation of published guidelines to harmonise practice through the development of standardised MRI protocols and reports in MS that are informative, yet feasible and adaptable to a range of clinical settings within the National Health Service (NHS).

Here we report the outcome of a workshop that brought together MS specialists geographically representing the UK NHS and with expertise in MRI and that identified clinically informative MRI protocols applicable in a variety of clinical settings, from district general hospitals to tertiary centres.

## Methods

The authors of this manuscript are MS specialists geographically representing the UK NHS and with expertise in MRI, either as neurologists with academic interest in neuroimaging or as neuroradiologists with MS as a specialist interest. They work as clinical academics and/or as NHS consultants in specialised MS centres, as well as in district general hospitals (DGH). Prior to the authors’ meeting to plan the present work, a comprehensive literature review and examination of MRI protocols was conducted to identify the points that made implementation of the existing guidelines challenging in the clinical practice across NHS settings. The authors completed a pre-meeting questionnaire to gather information on their experience with MRI protocols in NHS centres and to inform the scope for improvement. Both literature review and the outcome of the questionnaires were discussed in detail during the meeting to assess the need for a standardised MRI protocol applicable across clinical settings and to evaluate between-centre differences that could inform the development of minimum recommended MRI protocol for MS.

## Results

### Timing of MRI scans for MS diagnosis and monitoring

**Fig. 1:**
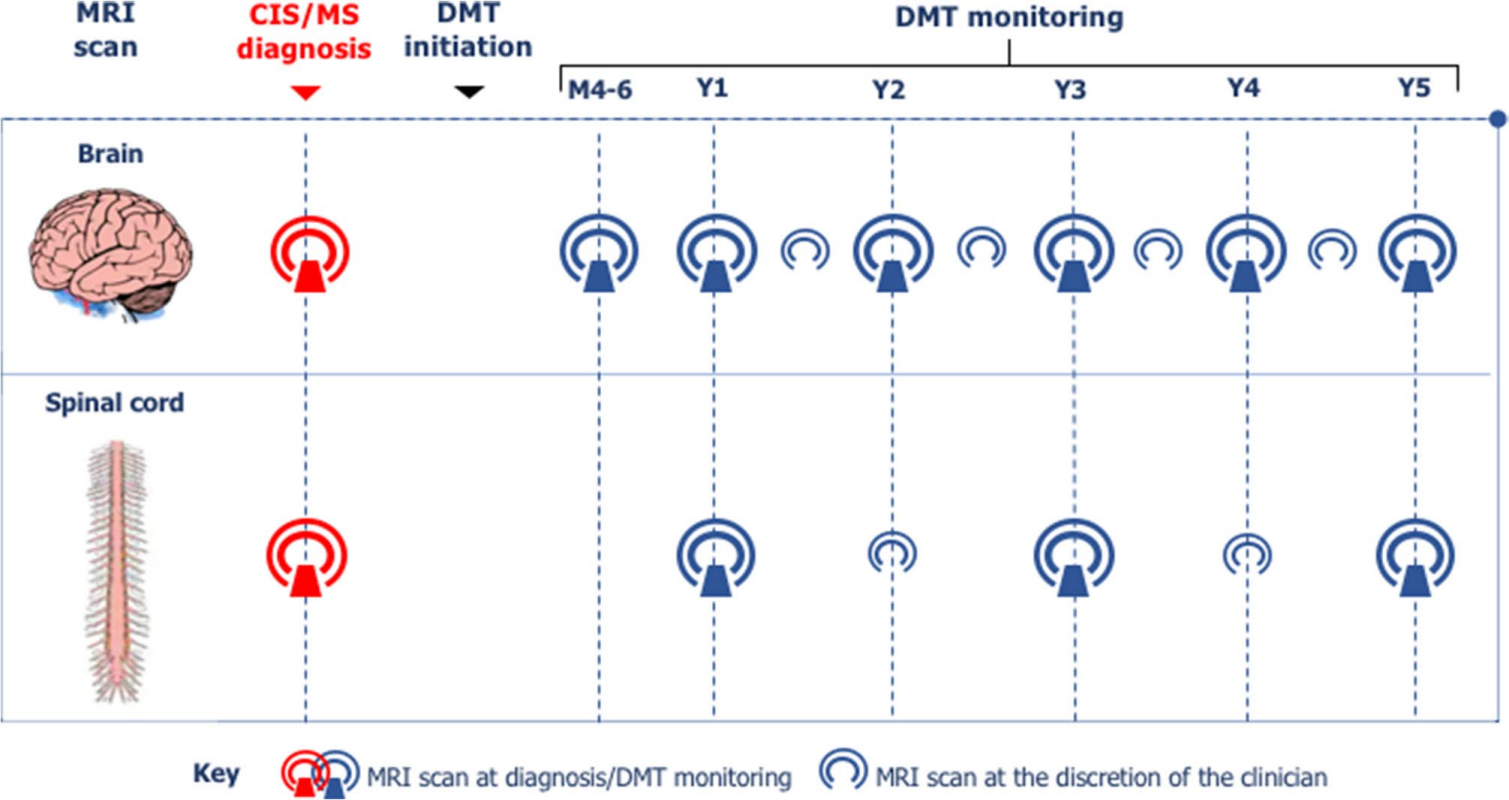
Timing of MRI scans in the diagnosis and monitoring of MS patients. The authors advised to perform a brain and spinal cord scan at diagnosis. After the beginning of DMT or after a change in DMT, brain MRI scan was indicated yearly for at least 5 years, with a re-baseline brain scan 4–6 months after initiating or changing DMT. This was to interpret more confidently response to treatment at an early stage of the disease. More frequent scans were left at the discretion of the clinician, based on individual cases. Spinal cord MRI scan was advised every 2 years for at least 5 years after initiation or change of DMT, even in the absence of clear spinal cord signs or symptoms, with more frequent scans left at the discretion of the clinician, based on individual cases. In patients with predominant involvement of the spinal cord, yearly spinal scans may be more appropriate. The initial assessments should be conducted with the use of contrast; subsequent MRI scans for monitoring of disease activity, as well as of DMT efficacy, may include contrast when clinically indicated*.* The proposed timing assumes the start of DMTs soon after diagnosis. When this is not possible or indicated, monitoring would be left at the discretion of the clinician, but advised to be regular. *CIS* clinically isolated syndrome, *MS* multiple sclerosis, *DMT* disease-modifying treatment, *MRI* magnetic resonance imaging

The clinical indications for MRI in MS vary from diagnosis to monitoring, as well as from assessment of treatment efficacy to surveillance for opportunistic infections (Table 1). An overview of the suggested timing for MRI scans in the diagnosis and monitoring of MS patients is reported for a period of 5 years in Fig. 1. This period is crucial for its long-term diagnostic and a prognostic value [10].

**Table 1:**
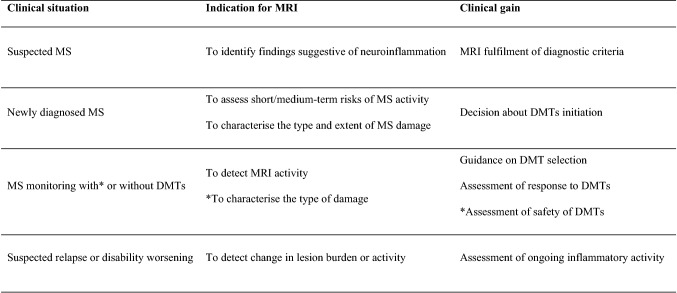
Indications for MRI in MS

The indication for MRI scanning in MS ranges from diagnosis to disease or DMTs monitoring, as highlighted below. MRI supports diagnosis, as well as disease monitoring. When a relapse cannot be confirmed purely on clinical grounds, MRI scan can be used to confirm clinical suspicion. Monitoring during DMTs aims to detect disease activity, as well as to characterise the type of damage that appears on the scan to exclude opportunistic infections such as PML

The asterisk indicates that MRI monitoring in patients on DMT is relevant to characterise the type of damage, as well as to assess safety of DMT

*DMT* disease-modifying treatment, *JVC* John Cunningham virus, *MRI* magnetic resonance imaging, *MS* multiple sclerosis, *PML* progressive multifocal leukoencephalopathy

#### Diagnosis

When the suspicion of clinically isolated syndrome (CIS) or of MS is raised, both brain and spinal cord scans should be requested [2, 11]. As well as providing information regarding lesion dissemination in space, spinal cord MRI is also valuable for differential diagnosis, when uncertainty exists over the nature of brain lesions [12, 13]. Although currently not routinely performed, MRI scanning of the optic nerve is important in patients who present with symptoms of visual dysfunction to aid the diagnostic process [2].

**Fig. 2:**
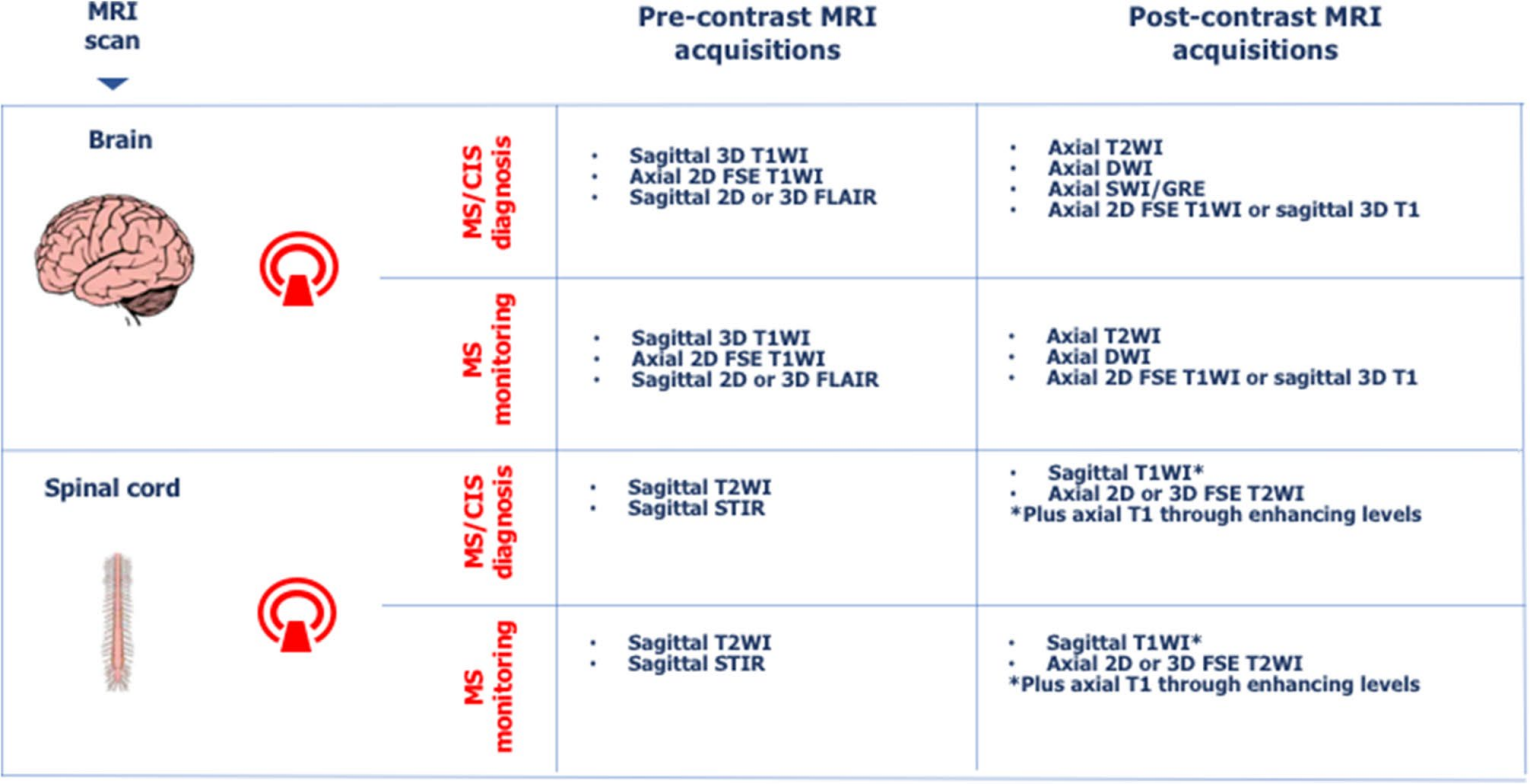
Suggested MRI acquisitions for MS diagnosis and monitoring. 3D acquisitions should be preferred as they make head positioning less critical, since readers can electronically re-slice the acquired image. *DIS* dissemination in space, *DIT* dissemination in time, *MRI* magnetic resonance imaging, *MS* multiple sclerosis, *CIS* clinically isolated syndrome, *2D* 2 dimensional, *3D* 3 dimensional, *T1WI* T1-weighted imaging, *T2WI* T2-weighted imaging, *FLAIR* fluid-attenuated inversion recovery, *STIR* short-TI inversion recovery, *DWI* diffusion-weighted imaging, *SWI* susceptibility-weighted imaging, *GRE* gradient recalled echo, *FSE* fast spin echo

The administration of a gadolinium-based contrast agent (Gd) is important in the diagnostic workup to demonstrate dissemination in time of lesions. Therefore, it should be included in the diagnostic MRI protocol, when clinical suspicion of CIS or MS has been raised [2]. Gd highlights focal inflammatory activity during the preceding weeks (typically 6–8 weeks) and thus aids earlier diagnosis. Post-contrast MRI scan is also useful for the purpose of differential diagnosis [14]. Figure 2 offers indications of a minimum set of clinically informative MRI acquisitions that could be considered in a MRI protocol for MS diagnosis. Other sequences, e.g. 3D-T2*echo-planar imaging (EPI) for central vein sign, double inversion recovery (DIR) for cortical lesions, phase-sensitive inversion-recovery (PSIR) for improved detection of spinal cord lesions and MR spectroscopy for atypically looking MS lesions (e.g., tumefactive lesions), may be also considered in addition, if possible and necessary. These sequences are more frequently adopted in specialist centres because they require longer acquisition time and physics expertise for standardisation.

#### Detection of disease activity and monitoring of efficacy

Monitoring MRI scans are useful in naïve patients, as well as at the beginning or following initiation or change of DMTs, or if a relapse is suspected, but cannot be confirmed purely on clinical grounds. Indeed, the importance of MRI to confirm stability is highlighted by the role of radiological activity in the evaluation of treatment response [1, 15].

Spinal cord MRI can reveal asymptomatic disease activity, as well as predict disease evolution [16]. Therefore, the authors felt that it should be performed regularly in addition to the brain scans, especially, but not exclusively, in patients in whom a previous involvement of the spinal cord has been demonstrated clinically or radiologically. Indeed, about 58% of new spinal cord lesions can be asymptomatic and 25% of patients with relapsing MS develops at least one asymptomatic spinal cord lesion over 1.5 years [11]; when only patients with stable RRMS are considered, 10% of them show subclinical spinal cord lesion activity alone [11]. Asymptomatic spinal cord lesions can also predict relapses, when combined with asymptomatic brain lesions [11]. Therefore, as spinal cord MRI can disclose subclinical disease activity in otherwise clinically stable MS, the authors supported its routine use in patient’s monitoring throughout the course of the disease and regardless of the clinical manifestation of MS in the cord.

Gd should be considered to aid detection of new inflammatory activity, when this would be too difficult to detect, due to high lesion burden, as well as for its prognostic utility, e.g., brain ring-enhancement patterns may forebode irreversible tissue damage and thus brain volume loss [17]. In the post-partum period, when monitoring is particularly important because of higher risk of relapse [18], the use of contrast enhancement may be limited by breastfeeding.

When patients are on specific DMTs, the primary purpose of a follow-up MRI scan is the detection of new inflammatory activity and the exclusion of opportunistic infections for safety of DMT prescription. As DMTs may reach their peak level of activity on MRI months after their initiation [19], a re-baseline scan beyond this time point is advisable. The authors felt that whether this scan should be performed with or without contrast enhancement should depend on clinical considerations such as the level of inflammatory activity at DMT initiation [20], the type of medication started [21] and the age of the patient [22] that all together define a risk for active disease. The presence or persistence of a Gd-active MRI scan at re-baseline could predict subsequent clinical and MRI activity [20, 23] and thus could indicate a subgroup of patients at higher risk of activity during DMTs.

The most recent classification of disease courses places importance on the presence or absence of disease activity also in the progressive forms of MS [24]. Also, with DMTs licenced in these stage (secondary progressive) or form (primary progressive) of the disease to control active inflammation, the authors felt that an approach to monitoring similar to the one used for relapsing MS would be reasonable, at least in the first few years (Fig. 1). MRI in progressive MS is a field in evolution that is likely to benefit from the introduction of methods to measure brain or cord atrophy in clinical practice [25].

#### Monitoring of DMTs safety

Patients treated with natalizumab or exposed to prolonged periods of lymphopenia with other DMTs may be at greater risk of progressive multifocal leukoencephalopathy (PML) [26]. Patients at risk of PML are typically those with anti-John Cunningham virus (JCV) antibodies, who have had treatment with natalizumab for over 18 months and have been given immunosuppressants before receiving natalizumab [27]. There is evidence to suggest that the management of PML is more successful if the condition is detected in a pre-symptomatic state through MRI [28]. Therefore, the differential diagnosis of new lesions is key: any lesions identified after initiating therapy with natalizumab, especially if more than 18 months, should be treated with suspicion. Any MRI changes consistent with PML that occur in immunocompromised patients should be classified as “radiologically-suspected PML” [29]. An algorithm has been developed to guide the use of MRI to monitor patients treated with natalizumab, based on anti-JCV antibody status and index value [29].

### MRI acquisition

The detailed definition of a standardised MRI acquisition protocol was beyond the scope of this work, as the authors felt that such protocol for implementation of MRI sequences in the NHS would be more appropriately defined and endorsed by relevant professional bodies. However, the authors identified some characteristics of the MRI acquisition protocol that would be informative and feasible in any clinical setting.

#### Scanner strength

Different clinical MRI scanners may use different magnetic fields of strength, with higher strengths yielding greater resolution. Thus, when scanning individual patients, changes in scanner strength may generate an inaccurate representation of disease evolution over time, over- or under-estimating MS lesions. Previous scanner strength should be a primary consideration when deciding about future MRI scans to ensure comparability between studies. When moving to a different scanner strength, especially from lower to higher, re-baseline scan is advisable. A minimum magnet strength of 1.5T (T) is important to preserve an adequate level of detail in the images and facilitate reproducibility between centres.

#### Sequences

MRI scanning requirements may differ from diagnosis to monitoring (Fig. 2), as well as from assessment of efficacy to surveillance of opportunistic infections. Among the MRI sequences typically helpful to detect MS lesions, some may be of particular help to gain insight into specific aspects of MS pathology, such as perivenular infiltration, cortical damage and tissue loss. Susceptibility weighted imaging (SWI) at diagnosis can demonstrate the perivenular orientation of lesions, which is helpful to distinguish between MS and ischemic lesions [30]. 3D-fluid attenuated inversion recovery (FLAIR) sequence is useful in diagnosis and monitoring of MS due to its ability to increase lesion’s visibility, as well as to detect cortical lesions [31]. The improved detection of posterior fossa lesions with 3D FLAIR might also obviate the need for axial T2 sequences [31]. 3D-T1 acquisition is useful for assessing changes in brain volume over time. While currently this parameter is likely to be qualitative in most of the reports, it may become quantitative, as the use of automated segmentation software becomes more widespread [32].

#### Head position

**Fig. 3:**
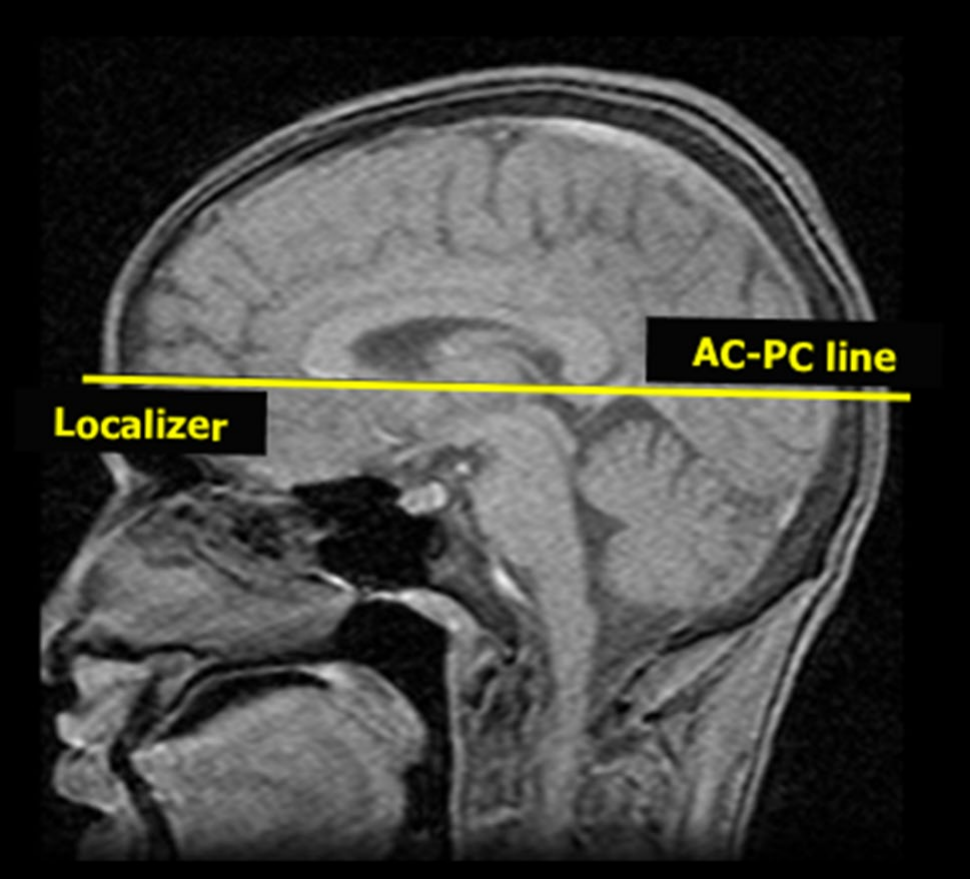
Head position during MRI acquisition. The anterior commissure–posterior commissure (AC–PC) line should be used to align head position to allow scan comparison, when only 2D acquisitions are possible. This line is typically ~ 9° steeper than the orbito-meatal line, traditionally used in computerised tomography (CT) scans. *MRI* magnetic resonance imaging, *2D* 2 dimensional

3D acquisitions mitigate the variability introduced by changes in head position by enabling readers to electronically re-slice the acquired image. However, this is not possible on all picture archiving and communication systems (PACS). Therefore, in this eventuality and, more importantly, for serial MRI scans using 2D acquisitions, the position of the patient within the magnet bore is important for between-study comparison. Differences in head position between scans can significantly change the orientation of axial images, making comparisons difficult. The use of the anterior commissure–posterior commissure (AC–PC) line, defined as the line passing through the superior edge of the AC and the inferior edge of the PC, for aligning head position remains recommended [33] (Fig. 3).

#### Use of contrast agent

Gd is frequently used for its ability to highlight regions of altered blood–brain barrier and thus areas of active inflammation, thereby providing an indicator of current disease activity and a means to assess DMTs efficacy and safety in PML surveillance [1, 34]. However, evidence suggests a retention of Gd in the body, following a scan [35]. Therefore, the Gd for routine MRI scans should be used when necessary, with specific macrocyclic Gd agents being recommended over linear agents for safety reasons [36, 37]. Since Gd may be used both at diagnosis and during DMTs or natural history monitoring, the authors felt that, in line with published recommendation, Gd should be used only when deemed to be clinically necessary: (1) at diagnosis, (2) in cases of high lesion burden to identify new lesions, (3) to confirm the clinical suspicion of disease activity (e.g., worsening in disability, for which a relapse is suspected, but no clear episode is identifiable), and (4) where it may critically inform treatment decisions (e.g., assessment of treatment efficacy [21] or safety [38]).

### MRI scan requests and reports

With an increasing number of scans being performed for MS diagnosis and management, there is further need to streamline scan requests and reports. Table 2 reports the proposed essential components of request forms and post-scan reports identified by the authors. Reports should be prepared with sufficient detail to stand alone, should the MRI images be unavailable. The use of the term “progression” on radiological reports was felt by the authors to be inaccurate and thus to be discouraged, with “evidence of radiologically active disease” or “radiological evolution” proposed as alternative terminology.

**Table 2:**
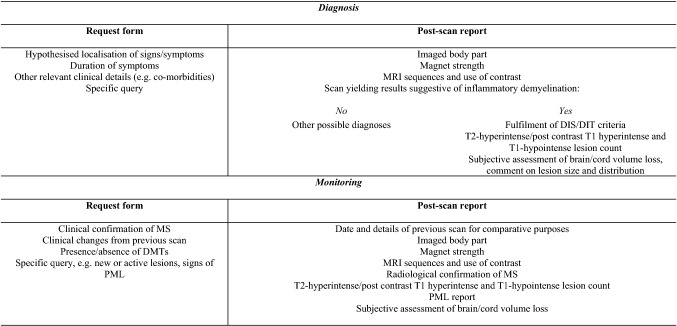
MRI request forms and post-scan reports

The authors suggested that the clinician provides the neuroradiologist with relevant information about the individual patient and his history (left). The post-scan reports should contain information that would allow interpretability of the results (right)

*DIT* duration in time, *DIS* duration in space, *MS* multiple sclerosis, *PML* progressive multifocal leukoencephalopathy, *DMTs* disease-modifying treatments, *MRI* magnetic resonance imaging

#### MS lesion count

Whenever possible, absolute lesion counts should be reported, i.e., information about the number of T2-hyperintense, T1-hypointense and T1-hyperintense lesions in the post-contrast T1-weighted scans. This is relevant for both diagnostic, as well as monitoring scans [1]. Where there is high lesion load, especially if lesions are confluent, the authors felt that, although not ideal, a categorical approach to the number of T2-hyperintense lesions would be acceptable (e.g., less than 9 T2-hyperintense lesions, between 10 and 20 hyperintense lesions etc.). For monitoring scans, the detection of new lesions and/or changes in the size of pre-existing lesions should be indicated. Lesion number should be indicated separately for the brain and the spinal cord. Indication of lesion location should be given to support the diagnostic process, as well as to provide clinically meaningful indications on the current or future patients’ disability [39].

#### Brain and spinal cord volume

Comments on brain or spinal cord volumes should be provided in the report, at least as qualitative evaluation of the presence of MS-related tissue loss, by comparing the last scan with what expected in the normal, age-matched population, or as an assessment of the rate of volume reduction over time, by comparing the last scan with the first (or the most meaningful) available scan for the same patient.

## Conclusions

In this report, we address the need for a standardisation of practice in the use of MRI for MS across clinical settings within the NHS. We suggest MRI scanning protocols and templates for MRI requests and post-scan reports in the attempt to facilitate diagnosis and optimise management of patients with MS, while considering the heterogeneity of resources and expertise that different clinical settings may present. The role of optic nerve imaging in MS was not discussed, as this is not currently included in the imaging diagnostic criteria for MS and, in MS monitoring, its informativeness is established by the clinician in each individual case [2].

Our proposed protocol aims to strike a balance between the need for frequent and accurate radiological information and the availability of equipment and staffing resources. The timing of MRI scans aims to capture, as early as possible, MS or its evolution that can be amenable to the starting or the changing of DMTs. The MRI acquisition protocol proposes a minimum, standardised set of meaningful information that can be used to manage MS. Indeed, published international guidelines offer guidance on MRI features that are most relevant to the diagnosis of MS [4]. Their implementation in clinical practice, however, has proven to be challenging across clinical settings, especially when the care of MS patients is outside specialist centres. Our proposed application of MRI for MS in clinical practice balances the specialist requirements with practical limitations imposed by limited resources by streamlining acquisition protocols and by standardising the timing of scans to integrate MRI findings in the clinical decision-making process. This effort can aid the impetus towards standardisation of practice and offer an example of research-informed service improvement to support optimisation of resources.

The authors also tackled the issue of effective communication between specialists, i.e., clinicians and radiologists, that is paramount in a multi-disciplinary context. We tailored our suggestions regarding the format of requests and reports for MRI scanning so that they can be used as a standardised, time-sparing tool for obtaining clinically relevant MRI information about MS patients. Indeed, the authors felt that clear, minimal information about the reason for the MRI scan was essential for the radiologist to correctly address the clinician’s question. Similarly, quantitative information about the MRI scan was felt to be necessary to manage MS effectively and promptly by clinicians. This quantitative, rather than merely descriptive, approach to MRI scan reporting for diagnostic, as well as for MRI monitoring scans, can contribute to an improved integration of MRI information in the clinical management of patients.
